# The Synergistic Effects of the Combination of L-Carnitine and Lycopene on the Lycopene Bioavailability and Duodenal Health of Roosters

**DOI:** 10.3390/ani13081274

**Published:** 2023-04-07

**Authors:** Wael Ennab, Nanwei Ye, Haoze Wu, Saif Ullah, Tavakolikazerooni Hadi, Anthony Pius Bassey, Sheeraz Mustafa, Jingle Jiang, Quanwei Wei, Fangxiong Shi

**Affiliations:** 1 National Experimental Teaching Demonstration Center of Animal Science, College of Animal Science and Technology, Nanjing Agricultural University, Nanjing 210095, China; 2 National Center of Meat Quality and Safety Control, Synergistic Innovation Center of Food Safety and Nutrition, College of Food Science and Technology, Nanjing Agricultural University, Nanjing 210095, China; 3 Faculty of Veterinary Animal Sciences, Ziauddin University (ZUFVAS), Karachi 75600, Pakistan; 4 Shanghai Endangered Species Conservation and Research Centre, Shanghai Zoo, Shanghai 200335, China

**Keywords:** lycopene, L-Carnitine, antioxidants combination, lycopene bioavailability, roosters

## Abstract

**Simple Summary:**

The objective of this study was to examine the effects of lycopene and L-carnitine in combination in the duodenum of roosters. The duodenum is important for Lycopene absorption, and the study found that the combination of Lycopene and L-Carnitine increased Lycopene absorption, resulting in higher levels of Lycopene in the serum. The combination also had positive effects on serum biochemistry, the expression of nutritional transport genes, and tight junction genes. These findings could provide useful insights for future research on the topic.

**Abstract:**

The objective of this study was to investigate the impact of Lycopene and L-Carnitine, individually or in combination, on various physiological and molecular factors related to intestinal health and absorption ability in Roosters, such as intestinal morphology, serum biochemical parameters, genes involved in Lycopene uptake, nutritional transport genes, and tight junction genes. The findings of the study revealed that the combination of L-Carnitine and Lycopene supplementation had been found to increase the serum concentration levels of TP and ALB. Interestingly, the relative mRNA expression of genes responsible for Lycopene uptakes, such as *SR-BI* and *BCO2*, was higher in the LC group compared to other groups. Additionally, the expression of specific nutritional transport genes in the duodenum was significantly affected by both CAR and LC supplementation groups. The tight junction gene *OCLN* showed a significant increase in expression in the combination group compared to using either Lycopene or L-Carnitine alone. This study concludes that using Lycopene and L-carnitine in combination in poultry feed can potentially improve intestinal morphology and serum biochemical parameters, increase Lycopene bioavailability, improve nutrients uptake, and enhance the integrity of duodenal tight junctions in Roosters.

## 1. Introduction

Over time, the global poultry industry has made remarkable progress in both quantity and quality. However, studies have shown that commercial poultry farming is vulnerable to various stresses, including technological, environmental, biological and nutritional factors [1,2]. Consequently, the free radicals overproduction resulting in oxidative stress is a widespread problem in commercial poultry production, as it is almost impossible to avoid these stresses altogether [3], which could lead to impair the endogenous antioxidant defense system, digestion process, nutritional transporters, and tight junctions proteins [4,5,6,7,8]. Accordingly, natural antioxidants should be included as an essential factor in poultry nutrition for enhancing immunity to increase production [3]. Antioxidants are widely used, and there are many studies demonstrating that antioxidants can play a protective role in animal production and reproduction, stress, immune system, antioxidant status, inflammation, disease prevention, and improve all over health [9,10,11,12,13].

Derived from an amino acid, L-Carnitine is a water-soluble product that facilitates the transportation of long-chain fatty acids to the mitochondria, where they go through oxidation to produce energy, and also helps in the removal of toxic compounds generated by this organelle to prevent their accumulation [14,15]. In addition to its essential role in lipid metabolism, L-Carnitine acts as a potent antioxidant and scavenges free radicals, which protects tissues from oxidative damage [16]. In addition to its importance in medicine, L-Carnitine has also gained considerable attention in poultry farming [17].

Recently, there has been growing scientific attention to the potential applications of Lycopene. It is a fat-soluble carotenoid belonging to the exogenous carotenoid family. The main absorption of lycopene occurs in the duodenum by passive or facultative diffusion and cleavage by *BCO1* and *BCO2* in enterocytes [18]. Additionally, it has been shown to exert notable antioxidant activity employing physical and chemical suppression (quenching) against singlet oxygen (^1^O_2_) by releasing energy in the form of heat against singlet oxygen (^1^O_2_), and it is considered the most efficient carotenoid for the inhibition of singlet oxygen [18]. Lycopene is also capable of maintaining oxidative homeostasis in the host organism through several mechanisms, including scavenging reactive oxygen species and regulating the production of antioxidant enzymes [19,20]. Despite all the studies showing the benefits of lycopene, the potential health benefits of Lycopene cannot be fully sufficiently exploited specifically in animal nutrition due to its low water solubility, chemical instability and low bioavailability, thereby reducing the protective roles and limiting its application [21]. In humans, more fat was used in the diet to increase the bioavailability of Lycopene, which is not healthy and not acceptable for most people in our modern time. Therefore, many studies have focused on aspects of human health, especially the use of Lycopene as an anti-cancer and fewer studies in animal science [22,23,24,25,26,27].

For antioxidants to influence/prevent oxidative damage caused by a particular reactive species, their antioxidant defense activity must increase sufficiently above normal levels, including endogenous levels, to affect oxidative damage [28]. However, the problem is that there are no superior antioxidants that can do everything individually [28]. At the same time, we need to keep up with our rapidly evolving world as the interest in natural antioxidants and improving their efficacy continues to grow, especially in poultry farms [29,30]. To overcome this problem, researchers in the last two decades resorted to a new old idea by combining antioxidants, which proved to be very effective [31,32,33,34,35].

Combining different antioxidants may be more effective than using them individually. For example, a combination of vitamin E and selenium may provide additional beneficial effects through their synergistic action on different levels of free radical production and lipid peroxidation [36]. They suggested that resveratrol combined with metformin might increase the antioxidant efficacy of metformin [31,32,33,34,35]. Recent studies and systematic reviews brought up the idea that a combination of two or three or more strong antioxidants can have an additive antidiabetic effect [37,38]. L-Carnitine was used in medicine and has been proven to improve the bioavailability of some drugs and supplements, such as sildenafil, losartan, gemcitabine, and chitosan-stearic acid [39,40,41,42]. Beta-carotene and lycopene are known as carotenoids; a previous study investigated the combination of L-carnitine and beta-carotene proved super therapeutic as an antioxidant effect [43].

Since the beneficial effect of lycopene depends on the ability of the duodenum to absorb the greatest amount of lycopene, and since we know that the combination of antioxidants can act synergistically to enhance the positive role in improving health [37,38], we chose this combination. In addition, L-carnitine was chosen for this combination because it has been repeatedly reported to improve the bioavailability of some substances, such as fatty acids [44], antioxidants [45], and drugs [42]. Moreover, the duodenum is susceptible to the risk of stress or inflammations, which can cause damage to the tissue, leading to various intestinal disorders. Therefore, the combination of antioxidants may be a valuable dietary strategy for improving the health and performance of roosters by enhancing the duodenum function. The aim of this study was, therefore, to investigate the effects of lycopene and L-carnitine in combination on the bioavailability of lycopene and the integrity of the duodenum. To date, there is limited information on the interaction of lycopene with other antioxidants [46,47,48,49]. To our knowledge, this is the first report investigating the combination of lycopene with L-carnitine

## 2. Materials and Methods

### 2.1. Animals and Treatments

A total of 32 Lijiang Roosters, 20 weeks old with similar original weights, were randomly assigned into 4 treatments with 8 replicates and 1 bird for each replicate. The whole experiment lasted for 8 weeks. The basal diet used in this study was according to the Nutrient Requirements of Poultry 1994, (Table 1) [50]. 4-treatment groups were divided as follows: (1) Control group where Roosters fed a basal diet (C); (2) Roosters fed a basal diet supplemented with 400 mg/kg Lycopene (LY); (3) Roosters fed a basal diet supplemented with 150 mg/kg L-Carnitine (CAR); (4) Roosters fed a basal diet supplemented with 400 + 150 mg/kg Lycopene and L-Carnitine in combination (LC). The supplemental levels of Lycopene and L-Carnitine were chosen according to previous studies [51,52,53,54]. Lycopene (purity ≥ 10%) was purchased from Millipore Sigma (Burlington, MA, USA), and it was incorporated into the diet in the form of an oily form containing 10% lycopene [55]. L-carnitine 98% was purchased from Sigma Technology (Danvers, USA). All birds were housed in level battery cages (dimension of each cage: 120 × 60 × 50 cm) in the Heying Poultry Breeding Company (Yancheng, China) with continuous light and controlled temperature. The access to water and feed was *ad libitum* for all roasters. The experiments were conducted under the Animal Care and Use Committee of Nanjing Agricultural University, Nanjing, China, with approval Numbers: 31572403 and 31402075.

### 2.2. Sample Collection

After 8 weeks of conducting the study, one bird was chosen from each replicate. Blood samples were taken from the wing vein and centrifuged at 3000× *g* for 15 min at 4 °C to obtain serum samples. These samples were then stored at −20 °C for further biochemical studies. The birds were humanely euthanized after blood collection. The duodenum was then carefully removed, and a segment of approximately 2 cm was enucleated from the center of the duodenum. The segments were carefully washed with phosphate buffer saline and immediately fixed in 4% PFA. The duodenal mucosa was carefully scraped with a microscopic glass slide and stored at −80 °C for analysis of gene expression and oxidative status.

### 2.3. Intestinal Morphology Analysis

The duodenum, jejunum, and ileum sections were fixed in 4% PFA for 24 h, followed by soaking in a graded series of ethanol and xylene before being embedded in paraffin. Using a Lecia RM2235 microtome (Leica Biosystems Inc., Buffalo Grove, IL, USA), the intestinal sections were cut to a thickness of 5 mm. The sections were then deparaffinized using xylene and gradually rehydrated with ethanol. HE staining was conducted following our laboratory’s protocol [56,57,58]. The images of the duodenum, jejunum and ileum were acquired using an Olympus simon-01 microscope (Olympus Optical Co., Ltd., Beijing, China). The values of villus height (VH) and crypt depth (CD) were measured 6 times from different villus and crypts per section from each broiler using the ImageJ software 2.9.0 (Fiji) [59].

### 2.4. Serum Lycopene Levels and Biochemical Indexes

The measurements of Albumin, total bilirubin, total protein, uric acid, and direct bilirubin were performed by using a commercial kit purchased from (Nanjing Jiancheng Bioengineering Institute, Nanjing, China) and the automatic clinical biochemistry analyzer (NVAS6805, NovaTech Co., Ltd., Changsha, China). All experimental procedures were accurately performed following the manufacturer’s instructions. The measurement of lycopene concentrations was according to a previous study [52].

### 2.5. Total RNA Extraction and mRNA Quantification

The total RNA of duodenal mucosae was extracted using an RNA isolator (Total RNA Extraction Reagent (R401-01)) (Nanjing Vazyme Biotech Co., Ltd., Nanjing, China). Using ND-2000 microspectrophotometer (Thermo Scientific, Wilmington, DE, USA) to identify the quality and concentrations of total RNA before the RNA reverse transcribed into complementary DNA using ABScript III RT Master Mix for qPCR with gDNA Remover Kit (RM21478, RM21479, ABclonal Technology, Woburn, MA, USA). The gDNA remover was added to remove the DNA, and a total of 1 mg of RNA was reverse-transcribed to complementary DNA. Complementary DNA was diluted to <50 ng gDNA or cDNA before real-time PCR. Real-time PCR was performed using the 2X Universal SYBR Green Fast qPCR Mix (ABclonal RK21203) reagent to carry out qPCR reaction on the QuantStudio 7 Real-Time PCR System (Thermo Scientific, Wilmington, DE, USA). The β-actin gene was selected to be the housekeeping gene to normalize the expression of the other target genes. The primers were synthesized by (Nanjing Vazyme Biotech Co., Ltd., Nanjing, China), and the primer sequences are shown in (Table 2). All genes were assayed 3 times. The reaction program was set as follows: stage 1:1 cycle at 95 °C for 3 min, then stage 2: was 40 cycles at 95 °C for 5 s, followed by 60 °C for 30 s. The melting curve was drawn according to the automatic instrument setting and used to verify the amplification of a single product. $2^{-∆∆ct}$ method was used to analyze the levels of relative gene expression after the normalization against β-actin.

### 2.6. Statistical Analysis

The Shapiro-Wilk test was used to assess the normality distribution of the data. Then one-way ANOVA analysis by Tukey’s post hoc tests. GraphPad Prism 7 software was used to analyze the data with multiple comparisons among groups (C, LY, CAR, LC). Data were presented as the mean and standard error of the mean. Differences were considered to be statistically significant at (*p* < 0.05).

## 3. Results

### 3.1. Intestinal Morphology

The effects of Lycopene and L-carnitine with/or without combination on the jejunal morphology of Roosters are shown in (Table 3). The data of duodenum and jejunum VH showed a significant increase in LY and CAR groups compared to the C group, while LC experimental group was significantly increased as compared to LY, CAR, and C groups (*p* < 0.05). Similarly, the experimental groups LY, CAR, and LC showed a significant increase in VCR of the duodenum compared to the C group (*p* < 0.05). Jejunum VH significantly increased in LY and CAR groups compared to the C group, and significance was found between LY and CAR groups (*p* < 0.05), while LC experimental group increased significantly compared to LY, CAR, and C groups (*p* < 0.05). Moreover, the experimental groups LY, CAR, and LC groups showed a significant increase of VCR in jejunum compared to the C group (*p* < 0.05). The supplementation of LY, CAR, and LC did not alter the VH, CD, and VCR in the ileum (*p* < 0.05). In contrast, our results agreed with a previous study and showed significance in VCR and VH of jejunum and duodenum but not in ileum when using Lycopene alone [60]. While the present study showed a significant increase in the jejunum and duodenum VH, CD, and VCR.

### 3.2. Relative mRNA Expression of Duodenal Related to Lycopene Uptake

**Figure 1:**
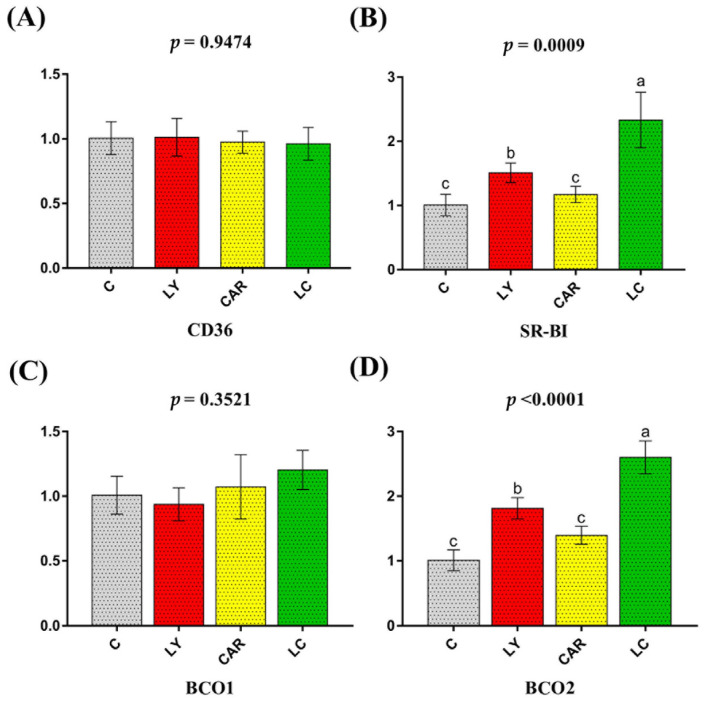
Relative mRNA expression of duodenal genes related to Lycopene uptake. (**A**), *CD36*; (**B**), *SR-BI*; (**C**), *BCO1*; (**D**), *BCO2*. Data are presented as mean value ± SEM (*n* = 3). Values without the same letter (a, b, c) represent statistically significant differences (*p* < 0.05). Abbreviations: C, Roosters fed a basal diet; LY, Roosters fed a basal diet supplemented with 400 mg/kg Lycopene; CAR, Roosters fed a basal diet supplemented with 150 mg/kg L-Carnitine; LC, Roosters fed a basal diet supplemented with (400 + 150) mg/kg Lycopene and L-Carnitine in combination.

The relative mRNA expression of duodenal genes related to lycopene uptake is displayed in Figure 1 and shows a significant difference in the expression of *SR-BI* and *BCO2* genes in LY and LC groups compared to C and CAR experimental groups (*p* < 0.05). At the same time, there were no significant differences in the expression of the *CD36* and *BCO1* genes in the duodenum among all experimental groups.

### 3.3. Relative mRNA Expression of Candidate Nutritional Transport Genes in the Duodenum

**Figure 2:**
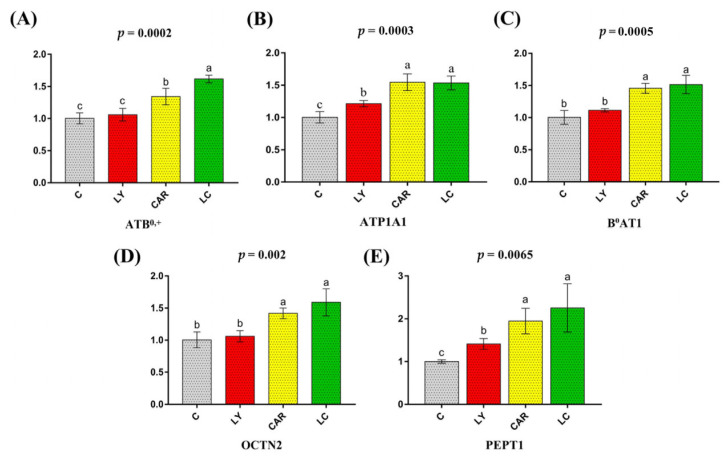
Relative mRNA expression of candidate nutritional transport genes in the duodenum. (**A**), *ATB^0,+^*; (**B**), *ATP1A1*; (**C**), *B^0^AT1*; (**D**), *OCTN2*; (**E**), *PEPT1*. Data are presented as mean value ± SEM (*n* = 3). Values without the same letter (a, b, c) represent statistically significant differences (*p* < 0.05). Abbreviations: C, Roosters fed a basal diet; LY, Roosters fed a basal diet supplemented with 400 mg/kg Lycopene; CAR, Roosters fed a basal diet supplemented with 150 mg/kg L-Carnitine; LC, Roosters fed a basal diet supplemented with (400 + 150) mg/kg Lycopene and L-Carnitine in combination.

Figure 2 displays the relative mRNA expression of nutritional transport genes in the duodenum. It reveals that the gene expression of *ATB^0,+^*, *ATP1A1*, *B^0^AT1*, *OCTN2*, and *PEPT1* showed a significant increase in the CAR and LC groups compared to the C and LY groups (*p* < 0.05). Additionally, the LY group had a significant increase in the *PEPT1* gene expression as compared to the C group (*p* < 0.05).

### 3.4. Serum Lycopene Levels

**Figure 3:**
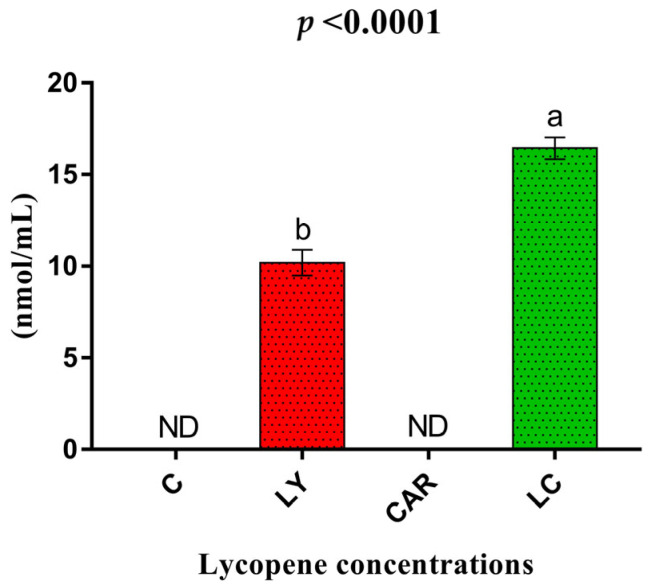
Serum Lycopene Levels. Data are presented as mean value ± SEM (*n* = 6). Values without the same letter (a, b, and ND) represent statistically significant differences (*p* < 0.05). Abbreviations: C, Roosters fed a basal diet; LY, Roosters fed a basal diet supplemented with 400 mg/kg Lycopene; CAR, Roosters fed a basal diet supplemented with 150 mg/kg L-Carnitine; LC, Roosters fed a basal diet supplemented with (400 + 150) mg/kg Lycopene and L-Carnitine in combination. (ND) pointed to not detected.

The effects of Lycopene levels in Roosters Serum are displayed in Figure 3. Our results reveal that the Lycopene levels were detected and showed a significant existence compared to the C and CAR groups (*p* < 0.05). Interestingly, the LC group had a significant increase in serum Lycopene levels compared to the LY group (*p* < 0.05). At the same time, the Lycopene was not detected in C and CAR groups.

### 3.5. Serum Biochemical Indexes

**Figure 4:**
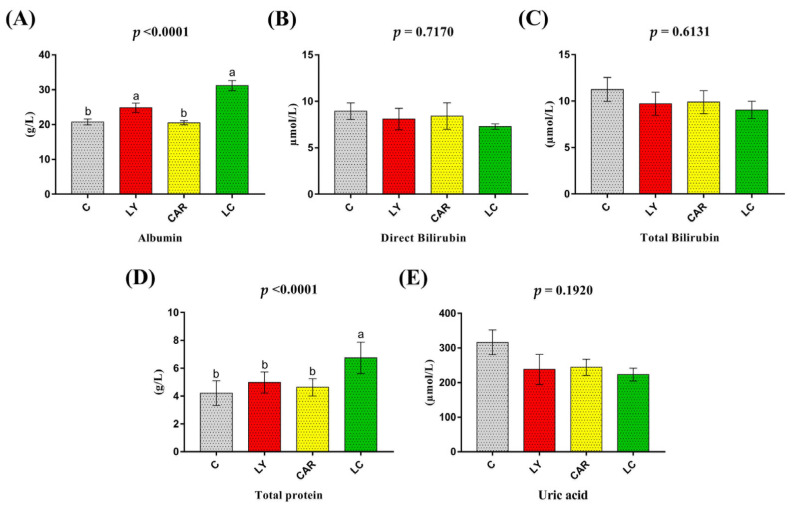
Effects of Antioxidants combination supplementations on the serum biochemical indexes of Roosters. (**A**), Albumin; (**B**), Direct Bilirubin; (**C**), Total Bilirubin; (**D**), Total protein; (**E**), Uric acid. Data are presented as mean value ± SEM (*n* = 8). Values without the same letter (a, b) represent statistically significant differences (*p* < 0.05). Abbreviations: C, Roosters fed a basal diet; LY, Roosters fed a basal diet supplemented with 400 mg/kg Lycopene; CAR, Roosters fed a basal diet supplemented with 150 mg/kg L-Carnitine; LC, Roosters fed a basal diet supplemented with (400 + 150) mg/kg Lycopene and L-Carnitine in combination.

The effects of Lycopene and L-carnitine with/or without combination on serum biochemical Parameters of Roosters at 28 weeks of age are shown in Figure 4. The Daily LC supplementation group significantly increased the concentrations of Albumin and Total protein compared with the other experimental groups (*p* < 0.05). However, the LY group showed some stability and a positive effect positively on serum biochemistry parameters, especially the concentration of Albumin, which increased significantly compared with CAR and C groups (*p* < 0.05). The supplementation of LY, CAR, and LC did not alter the concentrations of total bilirubin, direct bilirubin, and uric acid in the serum (*p* < 0.05).

### 3.6. mRNA Expression of Tight Junction Genes and Mucin in the Duodenum

**Figure 5:**
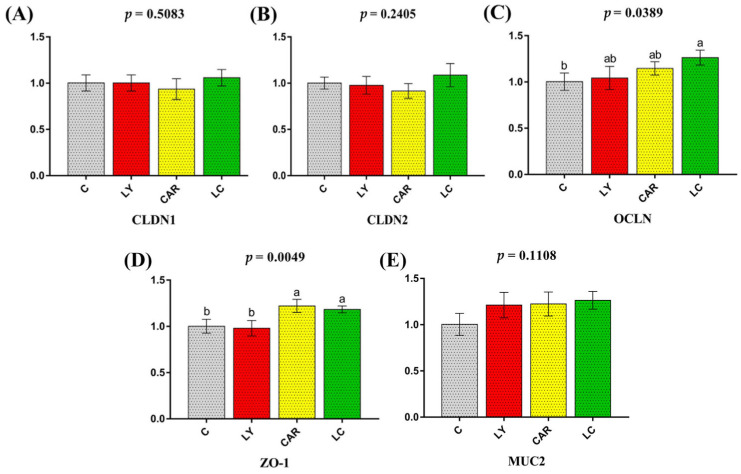
Relative mRNA expression of tight junction genes in the duodenum. (**A**), *CLDN1*; (**B**), *CLDN2*; (**C**), *OCLN*; (**D**), *ZO-1*; (**E**), *MUC2*. Data are presented as mean value ± SEM (*n* = 3). Values without the same letter (a, b) represent statistically significant differences (*p* > 0.05). Abbreviations: C, Roosters fed a basal diet; LY, Roosters fed a basal diet supplemented with 400 mg/kg Lycopene; CAR, Roosters fed a basal diet supplemented with 150 mg/kg L-Carnitine; LC, Roosters fed a basal diet supplemented with (400 + 150) mg/kg Lycopene and L-Carnitine in combination.

Relative mRNA expression of candidate nutritional transport genes in the duodenum is shown in Figure 5 and shows a significant increase of *OCLN* gene expression in the LC group compared to the C group (*p* < 0.05). At the same time, it was not significant among LY and CAR compared to C and LC groups. Moreover, *ZO-1* gene expression increased significantly in CAR and LC groups as compared to C and LY groups (*p* < 0.05).

## 4. Discussion

Human diets are abundant in Lycopene sources, such as tomatoes, carrots, ketchup, and others [61]. Unlike humans, the Lycopene sources in farm animals’ diets, such as chickens, are almost non-existent [62]. Involving Lycopene in the dietary diet is very important and valuable due to its antioxidant effects, as considered by [63,64]. Despite all the studies which proved the benefits of Lycopene, the potential health benefits cannot be well utilized due to its low water-solubility, chemical instability and low bioavailability, which increase the instability of a positive protective role [21]. So far, there is very little information on the interaction between lycopene and other antioxidants [49]. This led to a certain challenge in determining the dose for the present experiment. Previous publications have discussed in detail and reviewed various studies on lycopene and L-carnitine at different doses and have shown evidence of low toxicity even at high doses [65,66]. For this reason, and following previous studies that have shown good results with 400 mg/kg lycopene or 150 mg/kg L-carnitine, we have chosen the present dosages for the combination [51,52,53,54].

Intestinal integrity plays an important role in preventing the ingress of pathogens into chickens. The histological changes, such as VH, CD, and VCR, can potentially be used as an index for assessing digestion ability and intestinal function and add information to the assessment of the nutritional value of different types of chicken feed [67,68,69]. In agreement with previous studies, our results showed significance in VCR and VH of jejunum and duodenum but not in the ileum with adding Lycopene or L-Carnitine alone [67,70]. Moreover, the combination of Lycopene and L-Carnitine did not negatively affect intestinal morphology and showed a significant increase of VH and VCR in the duodenum and jejunum.

The small intestine is responsible for the absorption of lycopene, a carotenoid that is abundant in tomatoes and other red or pink fruits and vegetables. The amount of lycopene absorbed gradually decreases from the duodenum to the ileum, with the duodenum being the primary site for Lycopene absorption [71]. The intestinal cells play a crucial role in this process by incorporating lycopene into bile micelles and facilitating its transport through both passive diffusion and a cholesterol membrane transporter called *SR-BI* (scavenger receptor class B type I) [72]. Within the enterocytes, Lycopene undergoes cleavage by two enzymes, first is ß-carotene oxygenase 1 (*BCO1*), and the second is ß-carotene oxygenase 2 (*BCO2*), which produces apo-lycopene that is then exported into the portal blood, recent research has suggested that *BCO2* is primarily responsible for lycopene cleavage [73]. Studies have shown that inhibiting *SR-BI* impairs up to 60% of Lycopene uptake while overexpressing *SR-BI* in mice significantly increases the plasma lycopene concentration [74]. A previous study investigated the effects of Dietary L-carnitine enhances the lymphatic absorption of fat and α-tocopherol in ovariectomized rats [45]. They found that L-Carnitine may influence the process of lipid packaging and absorption by the enterocyte in rats and may explain in part the increased status of αTOH in L-Carnitine fed animals. In line with previous research and taking our results together [74,75,76,77], the present study has found that the expression of *SR-BI* and *BCO2* was higher in the duodenum, which may account for the higher concentration of Lycopene in the plasma.

The importance of carnitine carriers in various species has been highlighted, with the expression of transporters, such as *B^0^AT1*, *ATB^0,+^*, and *OCTN2,* being closely linked to increased uptake of carnitine [78,79,80]. L-Carnitine aids in the conversion of long-chain fatty acids to short-chain fatty acids for energy production, while *ATP1A1* uses Na^+^/K^+^ ATPase protein to transport charged atoms out of cells by utilizing energy from ATP [81,82]. Previous research has demonstrated that L-carnitine can increase ATP levels in the plasma [83]. PEPT1 plays a crucial role in nitrogen supply to the body by absorbing all proteins, such as tripeptides, dipeptides, or amino acids, by the small intestine, thereby increasing the active absorption of nutrient molecules, including lycopene [84]. A previous study found that the *B^0^AT1*, *ATB^0,+^,* and *PEPT1* expression is decreased due to infections causing significant pathology and resulting in decreased plasma carotenoid levels and body weight [85]. In addition, *ATP1A1* and *OCTN2* were associated with amino acid uptakes [80,86]. The current study found a significant increase in the expression of duodenum transporters, including *ATB^0,+^*, *ATP1A1*, *B^0^AT1*, *OCTN2*, and *PEPT1*. Taking the previous evidence and our results together makes us feel strongly that the role of amino acids in converting the long-chain fatty acids to short-chain fatty acids with micelles that conjugated the Lycopene substances might play a significant role in the Lycopene uptakes. In addition, the experimental group L-Carnitine and Lycopene in combination led to a significant increase in serum lycopene levels in line with increasing the expression of *SR-BI* and *BCO2*. However, further research is needed for a better understanding of these findings.

The serum biochemical parameters are a reflection of the physiological status of animals and are considered good indicators to diagnose nutritional problems and drug interaction in case of adverse effects, as noted in previous studies [87]. Lycopene has been shown to have immunomodulatory properties [88], and previous research on chickens has suggested that supplementing with Lycopene can increase the concentration levels of IgA and IgG [89]. Additionally, the combination of L-Carnitine and Lycopene supplementation has been found to increase the serum concentration levels of TP and ALB, indicating that it has the potential to enhance immunity and protein synthesis in broiler chickens. However, our results showed that dietary supplementation with either lycopene or L-carnitine alone did not significantly affect protein synthesis, except for an increase in ALB with lycopene supplementation and agreed with previous study [90]. Overall, our data suggest that the combination of lycopene and L-Carnitine may enhance protein synthesis in Roosters.

The tight junction proteins are important for maintaining the integrity and permeability of the intestinal barrier. They work by blocking the para-cellular space between epithelial cells, which prevents the diffusion of gut bacteria and other antigens across the epithelium [91]. Previous research has shown that the use of Lycopene alone can increase the expression of *CLDN1* but does not affect the expression of *OCLN* and *ZO-1* [60]. On the other hand, L-Carnitine supplementation alone can increase the expression of *ZO-1* [92]. The current study results showed that the combination of these two supplements could work synergistically and increase the expression of *OCLN* and *ZO-1* significantly in the duodenum. These findings suggest that the combination of Lycopene and L-Carnitine supplements can enhance the integrity and permeability of the intestinal barrier, which is crucial for preventing the diffusion of harmful substances across the epithelium. Overall, the study highlights the importance of Lycopene supplement in combination with L-Carnitine in improving the health and the functions of the duodenal barriers.

## 5. Conclusions

In conclusion, this study aimed to investigate the effects of a combination of lycopene and L-carnitine on lycopene bioavailability and duodenal integrity. The study provided evidence that a combination of lycopene and L-carnitine in the diet of Roosters can improve intestinal morphology, increase lycopene bioavailability, improve serum biochemical parameters, upregulate tight junction functions and increase nutrient transporter capacity. This is the first study to report on the potential effects of combining lycopene and L-carnitine, providing valuable insights and paving the way for future studies on this topic.

## Figures and Tables

**Table 1:** Ingredient composition and calculation of ingredients in the basal diet for rooster.

Ingredient (%)	20 to 28 Weeks
Corn	55.86
Soybean meal	19.10
Soybean oil	4.12
Wheat bran	15.12
NaCL	0.35
DL-methionine	0.15
Limestone	3.50
Dicalcium phosphate	1.52
Mineral premix	0.30
Vitamin premix	0.02
Choline chloride (50%)	0.23
Total	100
Calculation of nutrients	
Metabolizable energy MC/kg	12.05
Crude protein	14.5
Calcium	2.5
Methionine	0.32
Lysine	0.7
Available phosphorus	0.5

The following supplied per kg complete diet: nicotinic acid, 50 mg; biotin, 0.0325 mg; folic acid, 1.25 mg; niacin, 50 mg. vitamins: vitamin D3, 2500 IU; A, 12,500 IU; vitamin K, 2.65 mg; vitamin E, 80 mg; vitamin B2, 6 mg; vitamin B1, 2 mg; pantothenic acid, 12 mg; vitamin B6, 4 mg; vitamin B12, 0.025 mg; Fe, 80 mg; Mn, 100 mg; I, 0.35 mg; Cu, 8 mg; Se, 0.15 mg; and Co, 0.2 mg; Zn, 75 mg.

**Table 2:** Primer sequences were used for RT-qPCR in this study.

Gene Name	Sequences	Amplicon Size (bp)	Accession Number
*CLDN1*	Forward: GAGGATGACCAGGTGAAGAAGA Reverse: GCTGATCCAAACTCAAATCTGGTG	126	NM_001013611.2
*CLDN2*	Forward: ATCTCCAGCCATCTCTGTAACC Reverse: TCTCCCGCACGTTTACCTTT	150	NM_001277622.1
*OCLN*	Forward: AGCCCTCAATACCAGGATGTG Reverse: TGCTTCTTGCTTTGGTAGTCTG	83	NM_205128.1
*ZO-1*	Forward: AGGAGACCAAATTCTCAGGGTTA Reverse: AGACTCAACAATGCGACGATAAA	151	XM_040706827.1
*MUC2*	Forward: TACTCTTACCACCATAGTTACCAC Reverse: ATCAAACCACTCAGACCAATCAC	88	XM_040701667.1
*ATP1A1*	Forward: TTGAGCACTTTATCCACCTCATC Reverse: AGTGTCAGACATACCGTTACAG	175	NM_205521.1
*B^0^AT1*	Forward: TTTGGAACCCTAAATACGAGGAAT Reverse: CAATAATAGCATAGACCCAGCCA	75	XM_419056.7
*ATB^0,+^*	Forward: ATCGTCTACATTTACGGAGGAAAC Reverse: CCCATGTTGGATATAGCACTGA	196	XM_015278436.3
*PEPT1*	Forward: CAGACAGTCAACATCACTATGGG Reverse: GTCACATCTCCAGAACACTCATTA	203	NM_204365.1
*OCTN2*	Forward: GATTGAGGTTCCAGCCTACATTA Reverse: TAGATAGGGCACGAAGATGTGAA	143	NM_001045828.1
*CD36*	Forward: GGACCTTACACATACAGGGTCAG Reverse: GCATGTACGATATTGTGCCATTAG	79	NM_001030731.1
*SR-BI*	Forward: CAATGTTCATCTCCCACCCTC Reverse: GAATCTTCCCTGTTTGAAGGATACC	204	XM_415106.5
*BCO1*	Forward: TGGAGACACTAGATAAGGTAGAC Reverse: TGGTACAGAGGAAGGGATCTTA	164	NM_001364902.1
*BCO2*	Forward: TTGAGTTTGGTGAGGAGAAATACA Reverse: TGTGCTGGTTGTTAATCAGGTAG	138	XM_417929.7
*β-actin*	Forward: CATGGATGATGATATTGCTGCG Reverse: TACCAACCATCACACCCTGAT	140	NM_205518.1

Abbreviations: *ATP1A1*, ATPase Na^+^/K^+^ transporting subunit alpha-1; *B^0^AT*, neutral amino acid transporter; *ATB^0,+^*, solute carrier family 6 member 14; *PEPT1*, solute carrier family 15 member 1; *OCTN2*, solute carrier family 22 member 5; *CD36*, scavenger receptor class B member 3 (SCARB3); *SR-BI*, Scavenger receptor class B, type I; *BCO1*, beta carotene oxygenase 1; *BCO2*, beta carotene oxygenase 2; *CLDN1*, claudin 1; *CLDN2*, claudin 2; *MUC2*, mucin 2; *OCLN*, occludin; *ZO-1*, zonula occludens-1.

**Table 3:** Effects of Lycopene and L-Carnitine supplementation with/without combination on intestinal morphology of Roosters.

Items	Experimental Groups					
Duodenum	C	LY	CAR	LC	SEM	*p*-Value
VH (µm)	952.7 ^c^	1054 ^b^	1027 ^b^	1141 ^a^	14.5	<0.0001
CD (µm)	165.1	149.3	156.6	160.1	4.02	0.0955
VCR (µm)	5.787 ^b^	7.064 ^a^	6.574 ^a^	7.173 ^a^	0.16	<0.001
Jejunum						
VH (µm)	980.4 ^d^	1141 ^b^	1066 ^c^	1211 ^a^	15.24	<0.0001
CD (µm)	157.6	158.5	148	155.6	4.69	0.4162
VCR (µm)	6.25 ^b^	7.22 ^a^	7.22 ^a^	7.824 ^a^	0.22	<0.0008
Ileum						
VH (µm)	777.5	837.9	829.6	834.2	14.65	0.0360
CD (µm)	124.3	125.7	122.7	121	4.87	0.9159
VCR (µm)	6.295	6.705	6.827	6.925	0.24	0.3053

VH, villus height; CD, crypt depth; VCR, villus height-to-crypt depth ratio; C, Roosters fed a basal diet; LY, Roosters fed a basal diet supplemented with 400 mg/kg Lycopene; CAR, Roosters fed a basal diet supplemented with 150 mg/kg L-Carnitine; LC, Roosters fed a basal diet supplemented with 400 mg/kg Lycopene + 150 mg/kg L-Carnitine in combination; SEM, standard error of the mean; ^a–d^ Means (*n* = 6) with different letters within a row are significantly different (*p* < 0.05).

## Data Availability

Not applicable.
